# Supplementary material for: The long non-coding RNA βFaar regulates islet β-cell function and survival during obesity in mice
Source: Nat Commun. 2021 Jun 28;12:3997. doi: 10.1038/s41467-021-24302-6 (PMC8238983; doi:10.1038/s41467-021-24302-6)
Supplement: Supplementary file 1 — Supplementary Information [file 41467_2021_24302_MOESM1_ESM.pdf]

Supplementary Files

The long non-coding RNA βFaar regulates islet β-cell function and survival during obesity in mice

Fangfang Zhang[1, #], Yue Yang[1, #], Xi Chen[1], Yue Liu[1], Qianxing Hu[1], Bin Huang[1], Yuhong Liu[1], Yi Pan[1], Yanfeng Zhang[1], Dechen Liu[2], Rui Liang[3], Guoqing Li[2, 4], Qiong Wei[2, 4,*], Ling Li[2, 4,*], Liang Jin[1,*]

File list

Supplementary Figures:

Figure S1

Figure S2

Figure S3

Figure S4

Figure S5

Figure S6

Figure S7

Supplementary Spreadsheets:

Table S1

Table S2

Table S3

Table S4

Table S5

Table S6

Table S7

Supplementary Figures

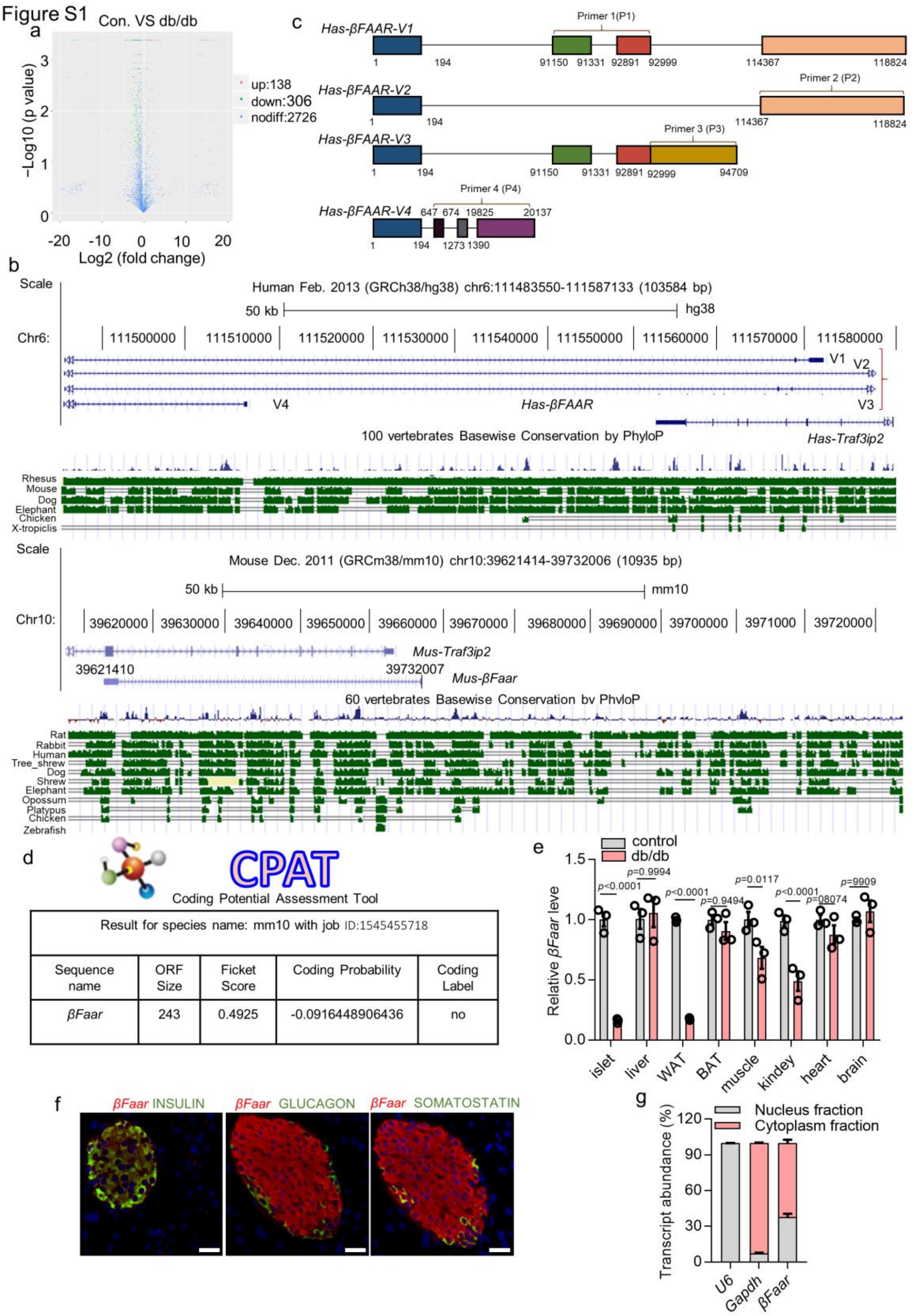

Figure S1 (a) The volcano of islet lncRNAs in db/db mice (red bar: up, green bar: down and blue bar: no different) versus control mice. (b) UCSC Genome Browser scheme of the human *βFaar*

variants 1–4 (red) and mouse *βFaar*. And the conservation between mouse *βFaar* and human *βFAAR* (Hg38 genome build). (c) Schematic diagram of primer design for detecting four variants of human *βFAAR*. (d) CPAT predicted the ability of *βFaar*-encoded proteins. (e) The expression levels of *βFaar* in different tissues of db/db mice compared to control mice (n=5). White adipose tissue (WAT), brown adipose tissue (BAT). (f) FISH analysis of *βFaar* expression level in the β cells, α cells and δ cells. The nuclei were stained with DAPI. Scale bar, 20 μm. (g) *βFaar* was enriched in the primary islet cell cytoplasm fraction of HFD mice. Levels of *βFaar*, *Gapdh* mRNA and *U6* small nuclear RNA in purified islet cell nuclear and cytoplasm fractions were detected by qRT-PCR. All qRT-PCR experiments above were performed in triplicates, and each group contained three batches of individual samples. The *p*-values by two-way ANOVA e is indicated. Data represent the mean ± SD. Source data are provided as a Source Data file.

Figure S2

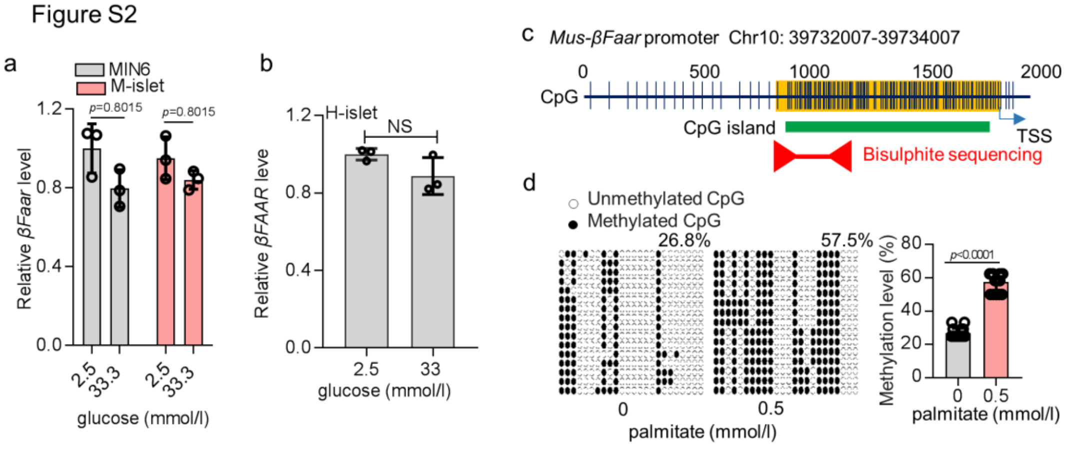

Figure S2 (a) MIN6 cells and primary islets were incubated with 2.5 mmol/l or 33.3 mmol/l glucose for 48 h and qRT-PCR was performed to examine the *βFaar* levels. (b) Human islets were incubated with 2.5 mmol/l or 33.3 mmol/l glucose for 48 h and qRT-PCR was performed to examine the *βFAAR* levels. (c) A schematic diagram of the CpG island and CpG sites in the *βFaar* promoter. Bisulfite Sequenced (BSP) regions are indicated by closed arrowheads and bars. (d) One

BSP regions of the *βFaar* promoter CpG island in the islet of MIN6 cells incubated with 0.5 mmol/l palmitate. Each box indicates the methylation status of the CpG site. Each row represents an individual sequenced DNA strand. Over 20 clones from each mixed sample were sequenced. The percentage of methylation in each sequenced region was indicated. All experiments above were performed in triplicates, and each group contained three batches of individual samples. The *p*-values by two-tailed unpaired Student's t test b and d, or two-way ANOVA a are indicated. Data represent the mean ± SD. Source data are provided as a Source Data file.

Figure S3

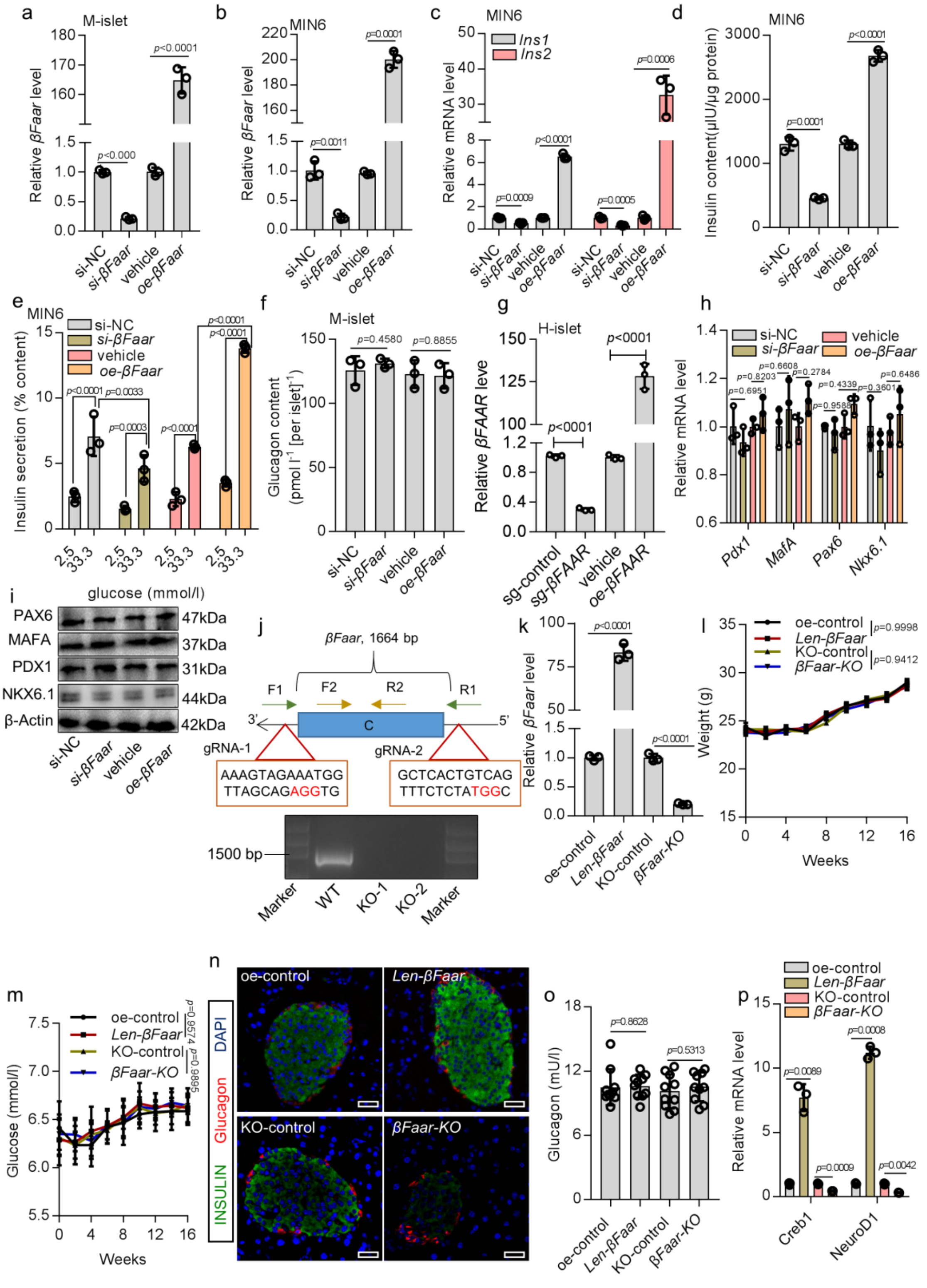

Figure S3 *βFaar* overexpression plasmid (*oe-βFaar*) and *βFaar* smart silence (*si-βFaar*) were transfected into primary islet cells and MIN6 cells for 48 h. Then the transfection efficiencies of *βFaar* were analyzed by qRT-PCR in primary islets (a) and in MIN6 cells (b). *βFaar*

overexpression plasmid (*oe-βFaar*) and *βFaar* smart silence (*si-βFaar*) were transfected into MIN6 cells for 48 h. Then qRT-PCR was performed to test the mRNA levels of insulin genes (*Ins1* and *Ins2*, c). Insulin content (d) and insulin secretion was analyzed by ELISA (e). (f) The glucagon level was measured by ELISA (e, n=5). (g)The expression levels of *βFAAR* in the human islets transfected with *oe-βFAAR* or *sg-βFAAR*. The mRNA (h) and protein (i) levels of PDX1, MAFA, PAX6 and NKX6.1. **(j)** Generation of *βFaar-KO* mice by CRISPR/Cas9. Knockout strategy with gRNA sequences (gRNA-1 and gRNA-2), and relative positions of primers (F1/R1) used for genomic PCR and primers (F2/R2) used for detecting deletions. (k) The expression levels of *βFaar* in the islets of *len-βFaar* mice and *βFaar–KO* mice. (l-m) The weight (l, n=10) and glucose (m, n=10) of *len-βFaar* mice and *βFaar–KO* mice. (n) Pancreatic sections were stained with insulin and glucagon antibody using immunofluorescence. Magnification: 20 x, Scale bars: 20 μm (n=3). (o) Glucagon content was tested by ELISA in the primary islet (n=9). (p) The mRNA levels of *Creb1* and *NeuroD1* in the islets of *len-βFaar* mice and *βFaar–KO* mice. All experiments above were performed in triplicates, and each group contained three batches of individual samples. The *p*-values by one-way ANOVA a, b, d, f, g, k and o or two-way ANOVA c, e, h, l-m and p are indicated. Data represent the mean ± SD. Source data are provided as a Source Data file.

Figure S4

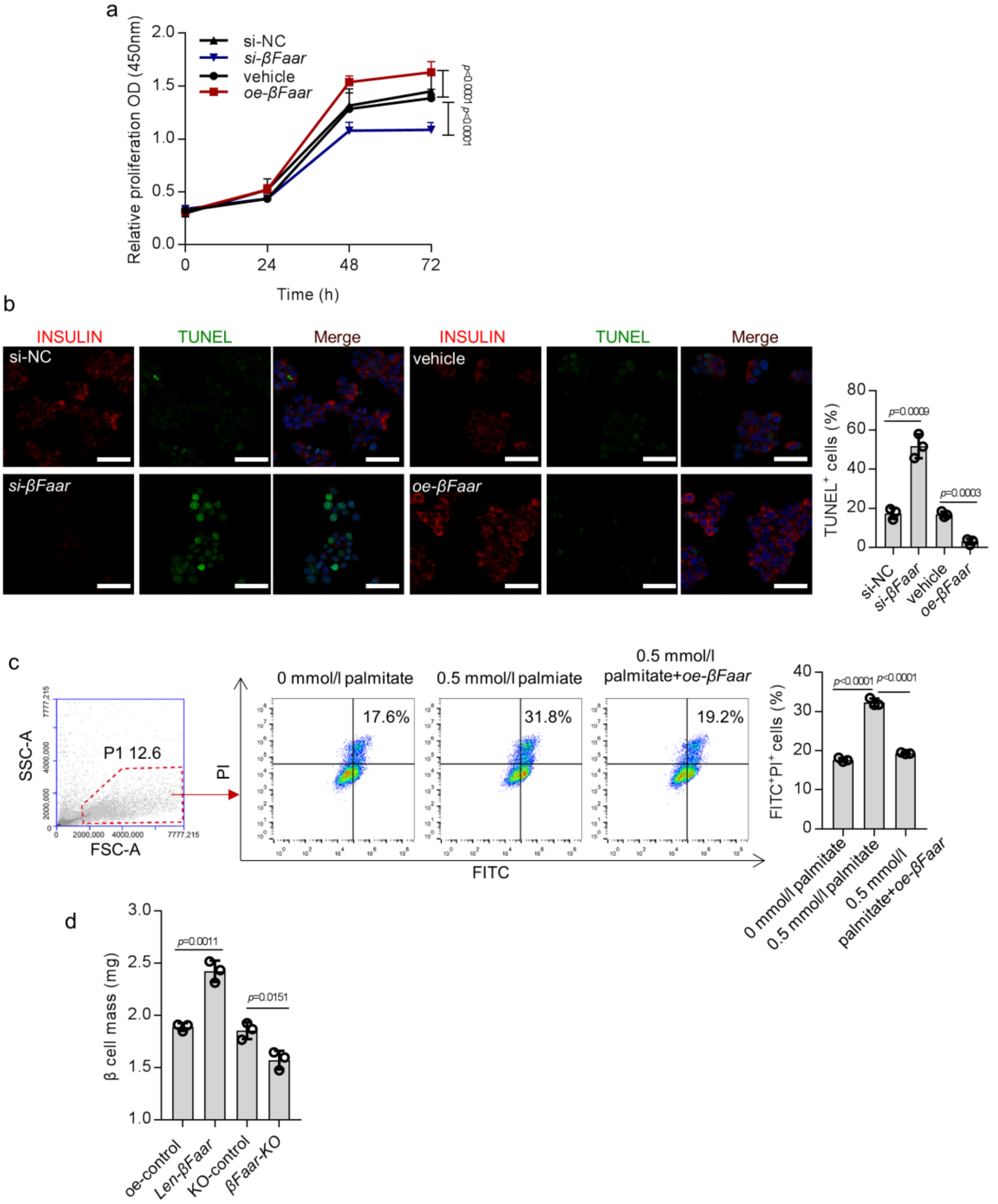

Figure S4 (a) *βFaar* overexpression plasmid (*oe-βFaar*) and *βFaar* smart silence (*si-βFaar*) were transfected into MIN6 cells, then CCk-8 assay was carried out at 24, 48, and 72 h after

transfection. (b) The both INSULIN and TUNEL positive cells were detected by immunofluorescence in the MIN6 cells, Magnification: 20 x, scale bar: 20 μm. (c) MIN6 cells were incubated with 0.5 mmol/l palmitate and co-transfected with *βFaar*, followed by Flow cytometry to examine the FITC+/PI+ cells. (d) The β-cell mass of *βFaar-KO* mice and len-*βFaar* mice was measured. All experiments above were performed in triplicates, and each group contained three batches of individual samples. The *p*-values by one-way ANOVA b-d, or two-way ANOVA a are indicated. Data represent the mean ± SD. Source data are provided as a Source Data file.

Figure S5

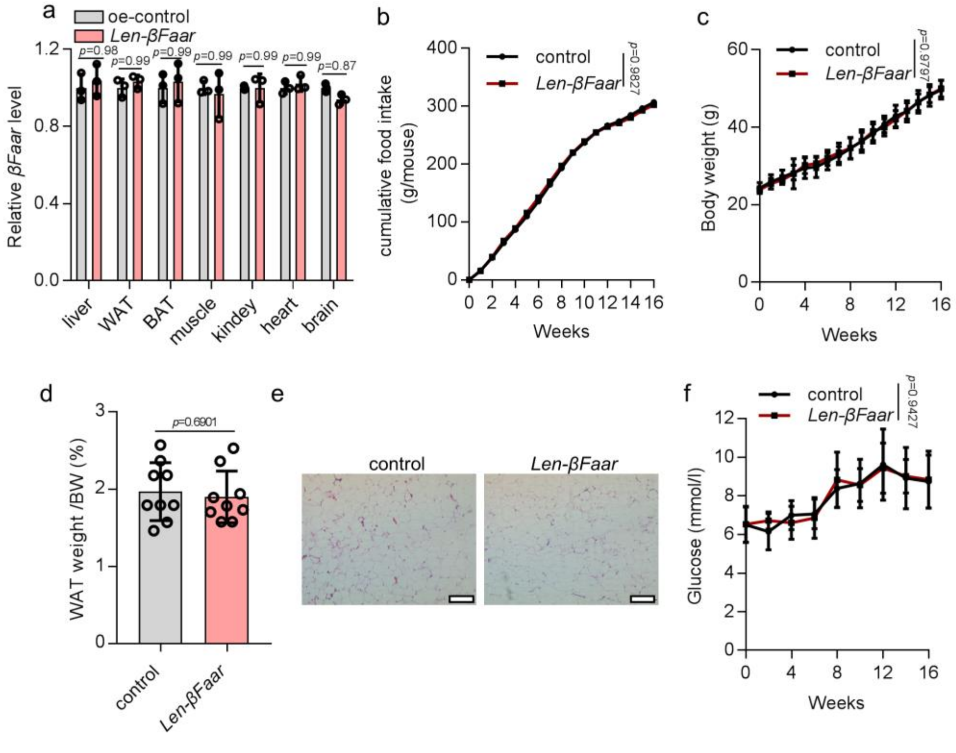

Figure S5 8-week-old male len-*βFaar* mice and control mice were exposed to HFD for 16 weeks. Then, the expression levels of *βFaar* in the different tissues were measured by qRT-PCR, White adipose tissue (WAT), brown adipose tissue (BAT, a), cumulative energy intake (b, n=10), changes in the body weight (c, n=10), white adipose tissue weight per body weight ratio (d, n=10)

and representative H&E staining of white adipose tissue (e, Magnification: 4 ×, scale bar: 100 μm, n=3). Fasting blood glucose levels (FBG) (f, n=10) were measured. All experiments above were performed in triplicates, and each group contained three batches of individual samples. The *p*-values by two-tailed unpaired Student's t test d, or two-way ANOVA a-c and f are indicated. Data file. Data represent the mean ± SEM. Source data are provided as a Source.

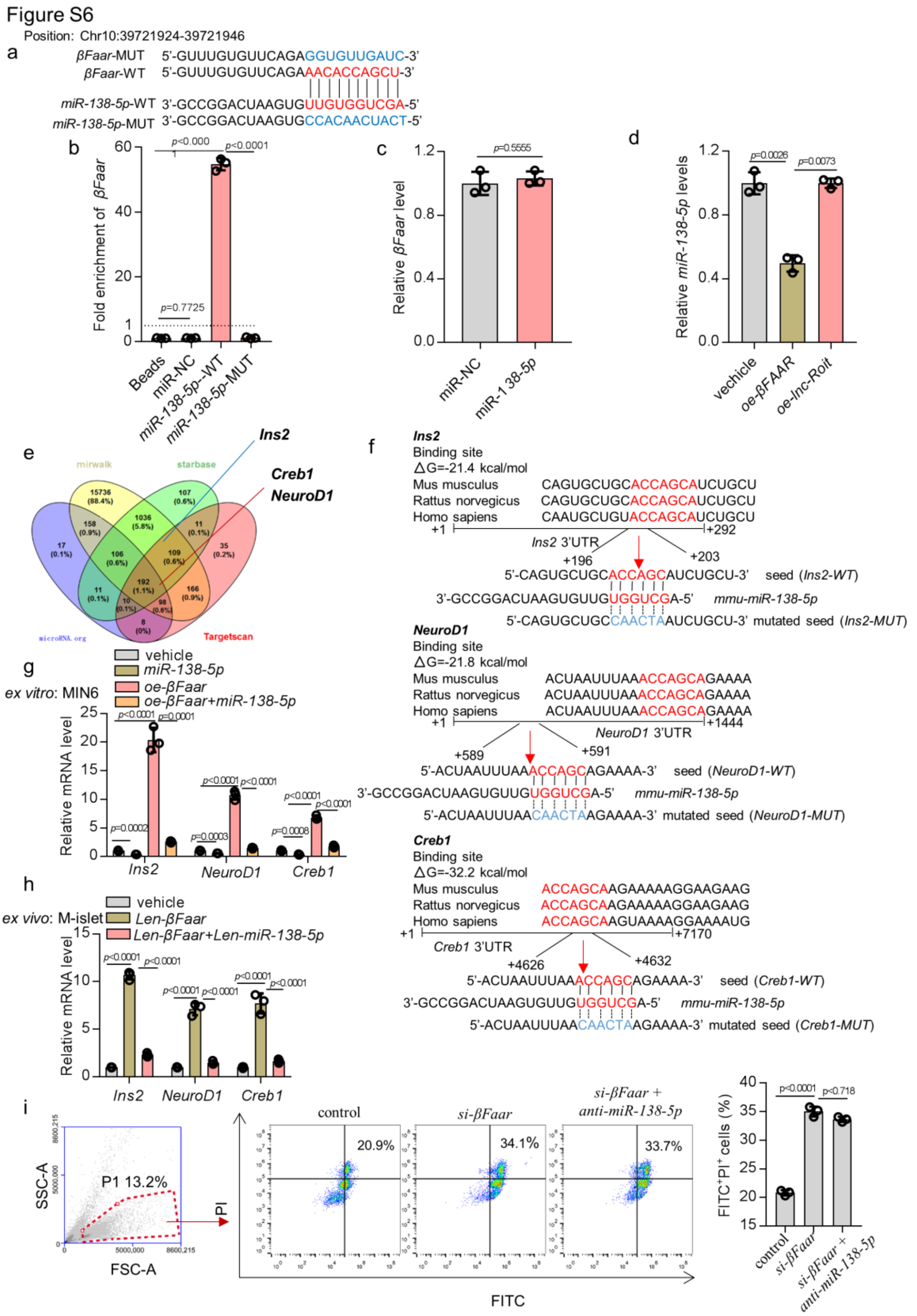

Figure S6 (a) The RNA algorithm predicted potential binding sites of *miR-138-5p* with *βFaar*, with considerable sequence complementary in the indicated regions. (b) MIN6 cell lysates were incubated with biotin-labeled *miR-138-5p*, after pull-down, *βFaar* was extracted and assessed by

qRT-PCR. (c) *miR-138-5p* was overexpressed in the MIN6 cells for 48 h, then qRT-PCR was to test the expression levels of *βFaar*. (d) *βFaar* or *lnc-Roit* was overexpressed in the MIN6 cells for 48 h, then qRT-PCR was to test the expression level of *miR-138-5p*. (e) Four independent miRNA target prediction algorithms were used to predict the target genes of *miR-138-5p*. (f) Graphic representation of the conserved *miR-138-5p* binding motif in the *Ins2*, *NeuroD1* and *Creb1* 3'UTR of three mammalian species. The schematic description of the wildtype (top) and mutated (bottom) *miR-138-5p* seed binding motif located in the murine *Ins2-*, *NeuroD1-* and *Creb1*-3'UTR used for transient reporter gene transfection experiments. (g) The mRNA levels of *Ins2*, *Creb1* and *NeuroD1* in the MIN6 cells transfected with *miR-138-5p* mimics or *βFaar* overexpression plasmid. (h) The mRNA levels of *Ins2*, *Creb1* and *NeuroD1* in the islets of *len-βFaar* treated mice or both len-*miR-138-5p* and *lent-βFaar* treated mice (n=5). (i) *miR-138-5p* mimics or *βFaar* overexpression plasmid (*oe-βFaar*) was transfected into MIN6 cells for 48 h. Then the $FITC^+/PI^+$ cells were measured by Flow cytometry. All experiments above were performed in triplicates, and each group contained three batches of individual samples. The *p*-values by two-tailed unpaired Student's t test c, one-way ANOVA b, d and i or two-way ANOVA g and h are indicated. Data represent the mean ± SD. Source data are provided as a Source Data file.

Figure S7

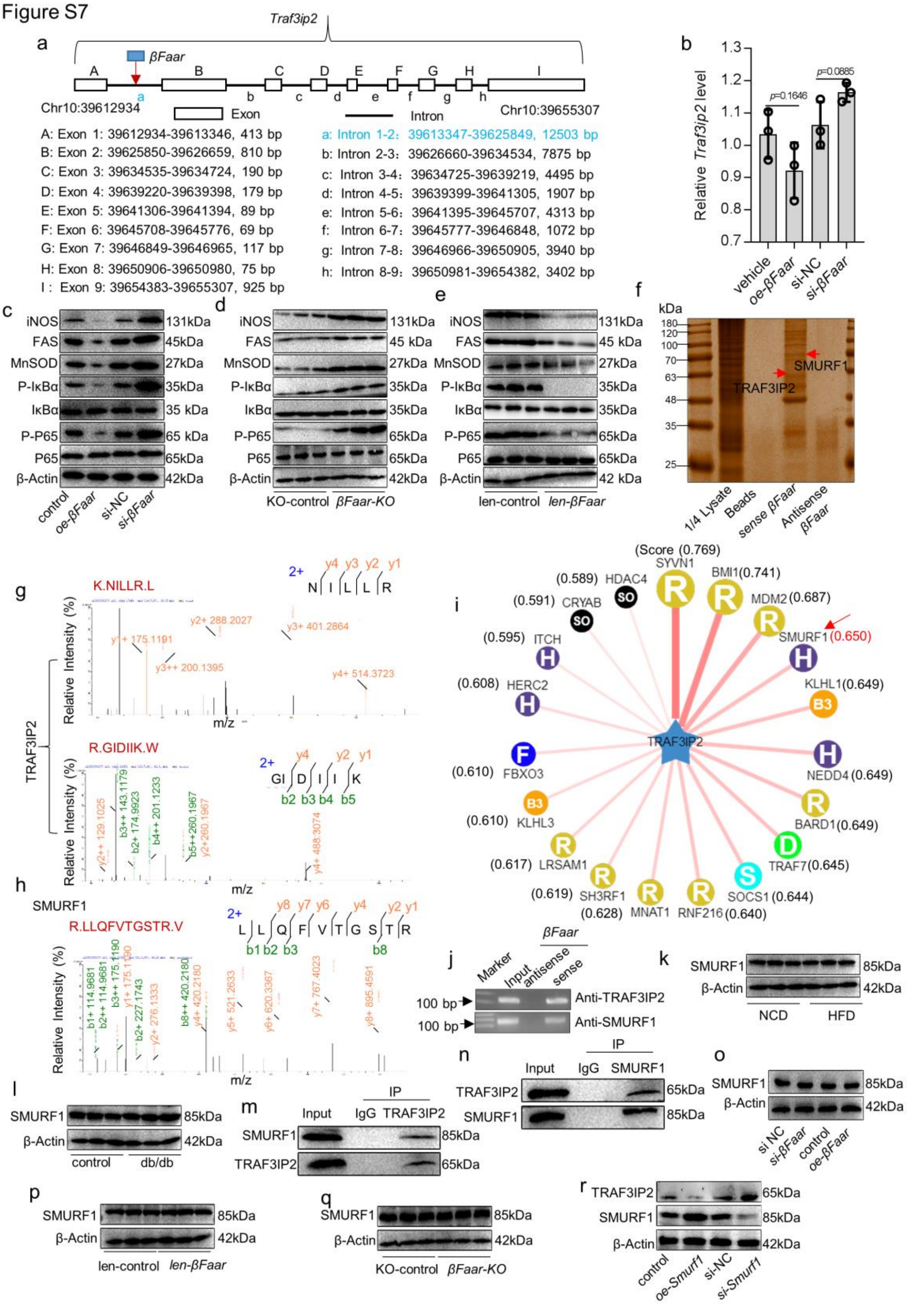

Figure S7 (a) Schematic illustration for the location of *βFaar* and TRAF3IP2. (b)The mRNA expression level of *Traf3ip2* in the MIN6 cells transfected with *oe-βFaar* or *si-βFaar*. The protein expression levels of P65, IκBα and some major NF-κB signaling targets (MnSOD, Fas, and iNOS)

in the MIN6 cells transfected with *oe-βFaar* or *si-βFaar* (c), in the islets of *βFaar-KO* mice (d, n=5) and in the islets of *len-βFaar* mice (e, n=5). (f) RNA pulldown assays were performed using biotin-labeled sense or antisense of *βFaar*. Coomassie brilliant blue staining was performed to identify the interacting proteins. TRAF3IP2 and SMURF1 was identified, as indicated by the red arrow. (g-h) Mass spectrometry (MS) profiles of the TRAF3IP2 (g) and SMURF1 (h) band retrieved by *βFaar*. The corresponding peptide sequences are listed on the top of the graphs. (i) The predicted primary E3 ligase for TRAF3IP2 in UbiBrowser database (http://ubibrowser.ncpsb.org/ubibrowser). (j) The MIN6 cell lysate was subjected to the anti-TRAF3IP2 or anti-SMURF1 RNA immunoprecipitation (RIP), and the precipitated RNAs were examined by PCR. (k-l) The protein levels of SMURF1 in the islets of HFD mice (k, n=5) and db/db mice (l, n=5). (m-n) MIN6 cells was subjected to co-immunoprecipitation with an antibody against immunoglobulin G (IgG), TRAF3IP2 (m) or IgG, SMURF1 (n) followed by western blotting using the indicated antibodies. The protein levels of SMURF1 in the MIN6 cells transfected with *si-βFaar* or *oe-βFaar* (o), in the islets of *len-βFaar* mice (p, n=5) and *βFaar-KO* mice (q, n=5). (r) Overexpression or knockdown of *Smurf1* in MIN6 cells, western blot results showed that SMURF1 negatively regulated TRAF3IP2 protein level. All experiments above were performed in triplicates, and each group contained three batches of individual samples. The *p*-values by one-way ANOVA b is indicated. Data represent the mean ± SD. Source data are provided as a Source Data file.

Supplementary Spreadsheets:

Table S1 Clinical characteristics of the control mice and db/db mice. The p-values by two-tailed unpaired Student's t test are indicated. Data represent the mean ± SD.

	control mice	db/db mice	*p*_valve
Number (male)	10	7	-
weeks (w)	10-11	11	-
body weight (g)	24.2±0.67	50.5±1.05	<0.0001
blood glucose (mmol/l)	6.4±0.59	25.4±0.71	<0.0001
Insulin content (ng/ml)	0.86±0.10	2.09±0.18	<0.001

Table S2 The protein binding partners for *βFaar*. Pull-down of antisense *βFaar* used as control.

Reference	PepCount	UniquePepCount	CoverPercent			
FileScan	Sequence	MH+	Diff(MH+)	Score	ExpectValue	Engine
tr|Q8N7N6|Q8N7N6_MOUSE E3 TRAF3-interacting protein 2						
$Q8N7N6-1	K.NILLR.L	628.41405	0.00012	27.21	9.51E-05	MASCOT
$Q8N7N6-2	R.GIDIIK.W	658.41339	-0.00008	21.25	3.75E-04	MASCOT
sp|Q9CUN6|SMUF1_MOUSE E3 ubiquitin-protein ligase SMURF1	R.LLQFVTGSTR.V	1121.63135	-0.0005	43.57	1.70E-03	MASCOT

Table S3 RNA islolated from islets of wide type mice and db/db mice, this table shows significantly changed lncRNA (Log2 (FPKM (db/db/control)) ≥2). The mean expression of each group was presented in a log2 scale. Cuffdiff (v2.2.1) provides statistical routines for determining differential expression in digital transcript or gene expression datasets using a model based on a negative binomial distribution. Transcripts or genes with corrected *p* values less than 0.05 and the absolute value of log2 (fold change) < 1 were assigned as significantly differentially expressed.

LncRNAID	Locus	log2(db_db/control)	up-or-down	*p*_value	*q*_value	LncRNAGeneID
TCONS_00036114	15:47800909-47858717	7.3737	up	0.00765	0.0359242	XLOC_014807
TCONS_00031953	14:32115304-32121204	4.33175	up	5.00E-05	0.000405964	XLOC_012832
TCONS_00020768	11:61690690-61698398	3.93045	up	5.00E-05	0.000405964	XLOC_007988
TCONS_00059507	2:169609973-169613216	3.86011	up	5.00E-05	0.000405964	XLOC_023426
TCONS_00112930	8:15519774-15520128	3.75208	up	5.00E-05	0.000405964	XLOC_043661
TCONS_00012300	10:19590964-19591700	3.60494	up	0.00075	0.00483592	XLOC_004882
TCONS_00113396	8:33713745-33719433	3.60229	up	5.00E-05	0.000405964	XLOC_043873
TCONS_00111640	7:128037640-128039087	3.58567	up	5.00E-05	0.000405964	XLOC_043261
TCONS_00025910	12:117345614-117348161	3.39313	up	5.00E-05	0.000405964	XLOC_009843
TCONS_00076113	4:127008521-127012532	3.35961	up	5.00E-05	0.000405964	XLOC_029793
TCONS_00066006	3:26531792-26536666	3.31637	up	5.00E-05	0.000405964	XLOC_025708
TCONS_00085743	5:135623618-135632108	3.10964	up	5.00E-05	0.000405964	XLOC_033375
TCONS_00110073	7:99805449-99807107	3.06053	up	5.00E-05	0.000405964	XLOC_042731
TCONS_00013978	10:99216402-99228852	3.05522	up	5.00E-05	0.000405964	XLOC_005549
TCONS_00032867	14:58257113-58257535	2.99624	up	0.0067	0.0321709	XLOC_013324
TCONS_00014026	10:107266774-107269021	2.90105	up	5.00E-05	0.000405964	XLOC_005579
TCONS_00039732	16:17132586-17133567	2.77091	up	5.00E-05	0.000405964	XLOC_016153
TCONS_00042918	16:90341508-90349872	2.71164	up	5.00E-05	0.000405964	XLOC_017276
TCONS_00040286	16:32200224-32201471	2.58761	up	5.00E-05	0.000405964	XLOC_016308
TCONS_00076508	4:134159390-134165301	2.53384	up	0.00625	0.0303596	XLOC_029910
TCONS_00028815	13:75789132-75790154	2.50052	up	0.00105	0.00651914	XLOC_011376
TCONS_00029687	13:21762871-21768348	2.46689	up	0.00265	0.0145602	XLOC_011782
TCONS_00104249	7:115164614-115165327	2.42252	up	0.0054	0.026827	XLOC_040491
TCONS_00045454	17:66016536-66020166	2.27467	up	0.0006	0.00397294	XLOC_018161
TCONS_00135076	Y:43039364-43039999	2.24887	up	0.00285	0.0154875	XLOC_053069
TCONS_00114520	8:81472874-81475054	2.21802	up	0.00545	0.0270462	XLOC_044323
TCONS_00126595	9:84365246-84366174	2.11796	up	0.00015	0.00113271	XLOC_048773
TCONS_00081338	4:149433490-149439207	2.08988	up	5.00E-05	0.000405964	XLOC_031723
TCONS_00124717	9:35043154-35044415	2.05419	up	5.00E-05	0.000405964	XLOC_048095
TCONS_00076505	4:134159390-134164570	2.05293	up	0.0074	0.0349499	XLOC_029910

TCONS_00022515	11:99153967-99154860	2.04468	up	0.0004	0.00276015	XLOC_008599
TCONS_00027782	13:14020278-14025509	2.03824	up	5.00E-05	0.000405964	XLOC_010836
TCONS_00110934	7:114303489-114310835	2.0254	up	5.00E-05	0.000405964	XLOC_043057
TCONS_00114529	8:81538027-81539235	2.02203	up	0.0094	0.0425947	XLOC_044332
TCONS_00040284	16:32150868-32153707	2.00741	up	5.00E-05	0.000405964	XLOC_016306
TCONS_00113292	8:26358987-26360676	-2.01447	down	5.00E-05	0.000405964	XLOC_043829
TCONS_00075446	4:110677777-110679987	-2.0172	down	5.00E-05	0.000405964	XLOC_029541
TCONS_00007142	1:123835223-123836678	-2.02724	down	0.00285	0.0154875	XLOC_002931
TCONS_00058390	2:141063434-141064984	-2.02741	down	0.0005	0.00337532	XLOC_023042
TCONS_00103218	7:91230867-91232277	-2.03655	down	0.0037	0.0193965	XLOC_040113
TCONS_00075456	4:110784897-110787216	-2.05077	down	0.0005	0.00337532	XLOC_029550
TCONS_00033736	14:11120570-11121359	-2.0525	down	0.00095	0.00596757	XLOC_013749
TCONS_00114341	8:72556891-72557446	-2.06929	down	0.00125	0.00759727	XLOC_044243
TCONS_00130974	X:169791681-169792078	-2.10691	down	0.01105	0.0487398	XLOC_050726
TCONS_00042288	16:37227614-37228559	-2.11649	down	0.0022	0.0123691	XLOC_017024
TCONS_00086729	5:7291257-7295079	-2.12271	down	5.00E-05	0.000405964	XLOC_033701
TCONS_00024485	12:41250721-41252027	-2.12744	down	0.0082	0.0380559	XLOC_009268
TCONS_00089001	5:101574968-101577936	-2.13165	down	0.0002	0.00147469	XLOC_034624
TCONS_00120980	9:46634190-46635325	-2.13286	down	5.00E-05	0.000405964	XLOC_046848
TCONS_00086732	5:7335992-7342284	-2.13778	down	5.00E-05	0.000405964	XLOC_033704
TCONS_00129625	X:94736314-94737168	-2.14339	down	0.0051	0.0255441	XLOC_050048
TCONS_00117138	8:38230571-38242857	-2.15932	down	5.00E-05	0.000405964	XLOC_045298
TCONS_00020983	11:69010563-69012465	-2.16278	down	5.00E-05	0.000405964	XLOC_008064
TCONS_00086725	5:7262632-7264765	-2.17615	down	0.0004	0.00276015	XLOC_033697
TCONS_00038684	15:83295015-83295639	-2.19586	down	0.0006	0.00397294	XLOC_015790
TCONS_00007130	1:123591091-123594749	-2.1964	down	5.00E-05	0.000405964	XLOC_002921
TCONS_00117139	8:38244483-38247173	-2.20473	down	5.00E-05	0.000405964	XLOC_045299
TCONS_00035667	14:124608754-124609771	-2.21047	down	0.0021	0.011887	XLOC_014589
TCONS_00033756	14:12755108-12779331	-2.21484	down	0.003	0.0161718	XLOC_013758
TCONS_00022141	11:93096002-93099149	-2.22143	down	0.0001	0.000776767	XLOC_008473
TCONS_00007138	1:123798087-123801483	-2.23628	down	0.0003	0.00213072	XLOC_002927
TCONS_00075453	4:110747566-110750110	-2.24045	down	0.00015	0.00113271	XLOC_029547
TCONS_00029604	13:18473163-18473549	-2.25241	down	0.00205	0.0116398	XLOC_011739
TCONS_00117388	8:54515485-54516954	-2.2583	down	5.00E-05	0.000405964	XLOC_045416
TCONS_00120984	9:46677337-46677853	-2.25834	down	0.00135	0.00812612	XLOC_046850
TCONS_00117145	8:38368437-38413473	-2.26085	down	5.00E-05	0.000405964	XLOC_045305
TCONS_00132669	X:105279925-105280729	-2.26798	down	0.00015	0.00113271	XLOC_051562
TCONS_00093063	6:61971462-62035112	-2.26808	down	5.00E-05	0.000405964	XLOC_036009
TCONS_00082337	5:29546120-29547009	-2.26851	down	0.0045	0.022931	XLOC_032125
TCONS_00082332	5:29498201-29498669	-2.27312	down	0.0102	0.0455858	XLOC_032120
TCONS_00026741	12:83458232-83459304	-2.2753	down	0.0001	0.000776767	XLOC_010246
TCONS_00035671	14:124661213-124662674	-2.27809	down	0.00095	0.00596757	XLOC_014593
TCONS_00044996	17:42890031-42895578	-2.28247	down	5.00E-05	0.000405964	XLOC_017975

TCONS_00007143	1:123839005-123840272	-2.31041	down	0.00665	0.0319726	XLOC_002932
TCONS_00074884	4:89313202-89313770	-2.32905	down	0.00695	0.0331516	XLOC_029352
TCONS_00117153	8:38607779-38659620	-2.33758	down	5.00E-05	0.000405964	XLOC_045309
TCONS_00073086	3:142126576-142127208	-2.33948	down	0.00215	0.0121301	XLOC_028525
TCONS_00074372	4:49072846-49073720	-2.34347	down	0.0003	0.00213072	XLOC_029058
TCONS_00117144	8:38365466-38368127	-2.38719	down	5.00E-05	0.000405964	XLOC_045304
TCONS_00086727	5:7269717-7273653	-2.41841	down	5.00E-05	0.000405964	XLOC_033699
TCONS_00044797	17:35994512-36001107	-2.42134	down	5.00E-05	0.000405964	XLOC_017874
TCONS_00094693	6:118990303-119001334	-2.42613	down	5.00E-05	0.000405964	XLOC_036644
TCONS_00075466	4:111375333-111384873	-2.4529	down	5.00E-05	0.000405964	XLOC_029558
TCONS_00117134	8:38161331-38162270	-2.46761	down	5.00E-05	0.000405964	XLOC_045294
TCONS_00004692	1:8965831-8969198	-2.47587	down	0.0002	0.00147469	XLOC_001902
TCONS_00131666	X:49960846-49966826	-2.48961	down	5.00E-05	0.000405964	XLOC_051096
TCONS_00117141	8:38256250-38267852	-2.49116	down	5.00E-05	0.000405964	XLOC_045301
TCONS_00005334	1:37821478-37826821	-2.49361	down	5.00E-05	0.000405964	XLOC_002199
TCONS_00046096	17:22806489-22809026	-2.50123	down	5.00E-05	0.000405964	XLOC_018471
TCONS_00117136	8:38201072-38202154	-2.51827	down	5.00E-05	0.000405964	XLOC_045296
TCONS_00074819	4:86725461-86726373	-2.53331	down	5.00E-05	0.000405964	XLOC_029308
TCONS_00002698	1:124357074-124357752	-2.57228	down	0.003	0.0161718	XLOC_001101
TCONS_00109037	7:61753391-61932925	-2.62226	down	5.00E-05	0.000405964	XLOC_042322
TCONS_00120985	9:46689374-46690161	-2.63063	down	0.00075	0.00483592	XLOC_046851
TCONS_00016304	11:69378582-69379234	-2.67155	down	0.0003	0.00213072	XLOC_006461
TCONS_00075467	4:111385036-111386273	-2.73568	down	0.0052	0.0259576	XLOC_029559
TCONS_00007129	1:123579970-123583373	-2.76055	down	5.00E-05	0.000405964	XLOC_002920
TCONS_00117137	8:38225227-38229206	-2.77721	down	5.00E-05	0.000405964	XLOC_045297
TCONS_00038346	15:76983028-76983664	-2.78953	down	0.00595	0.0291388	XLOC_015663
TCONS_00025176	12:86990956-86991634	-2.79751	down	0.0033	0.0175705	XLOC_009548
TCONS_00030868	13:87374242-87374701	-2.85348	down	0.0049	0.0247019	XLOC_012349
TCONS_00037465	15:103609624-103631620	-2.94568	down	5.00E-05	0.000405964	XLOC_015300
TCONS_00014050	10:110074710-110075643	-2.96648	down	0.00525	0.0261763	XLOC_005593
TCONS_00103204	7:90967381-90968035	-2.98033	down	0.00105	0.00651914	XLOC_040101
TCONS_00068830	3:125439932-125440329	-3.09221	down	0.00055	0.00367813	XLOC_026752
TCONS_00007127	1:123553792-123555886	-3.11014	down	0.003	0.0161718	XLOC_002918
TCONS_00033765	14:13907755-13908131	-3.11271	down	0.00905	0.0412702	XLOC_013763
TCONS_00085072	5:120734967-120736829	-3.15994	down	0.00315	0.0168747	XLOC_033162
TCONS_00121638	9:65245718-65313543	-3.17022	down	5.00E-05	0.000405964	XLOC_047064
TCONS_00117152	8:38547119-38607650	-3.17865	down	5.00E-05	0.000405964	XLOC_045309
TCONS_00046086	17:22066777-22078614	-3.21878	down	0.00035	0.0024492	XLOC_018464
TCONS_00037466	15:103633492-103637055	-3.33914	down	5.00E-05	0.000405964	XLOC_015301
TCONS_00037467	15:103663225-103664522	-3.51759	down	0.00025	0.00180921	XLOC_015302
TCONS_00014051	10:110096872-110097598	-3.74226	down	0.0065	0.0313967	XLOC_005594
TCONS_00014049	10:110068945-110070066	-3.79566	down	0.00455	0.0231451	XLOC_005592
TCONS_00012594	10:39621412-39622728	-3.9687	down	5.00E-05	0.000405964	XLOC_005032

TCONS_00080417	4:131641569-131656326	-4.09426	down	5.00E-05	0.000405964	XLOC_031400
TCONS_00028518	13:55184447-55190824	-4.19937	down	5.00E-05	0.000405964	XLOC_011221
TCONS_00091281	5:147265621-147269924	-4.2547	down	5.00E-05	0.000405964	XLOC_035292
TCONS_00020551	11:59197688-59202385	-4.3311	down	5.00E-05	0.000405964	XLOC_007920
TCONS_00017502	11:87992127-88006927	-4.65296	down	0.0045	0.022931	XLOC_006830
TCONS_00033723	14:10881748-10883042	-4.76966	down	0.0096	0.043344	XLOC_013736
TCONS_00109060	7:61985829-62085310	-4.77275	down	5.00E-05	0.000405964	XLOC_042331
TCONS_00033266	14:79844455-79965039	-5.42694	down	0.0114	0.0499873	XLOC_013484
TCONS_00080971	4:141206659-141210936	-8.73866	down	0.00435	0.0222987	XLOC_031576

Table S4 The primers used in PCR and DNA methylation (5’-3’).

Gene	Forward Primer	Reverse Primer
oe-βFaar	AGAACATTTCTCTATCGATACGAGG AGGGGGTTCTCCC	ACCAACAGTACCGGAATGCCATA CACATAAAACATATTTTTATTTTTT AGAAGG
sense-*βFaar*	TAATACGACTCACTATAGGGCGAG GAGGGGGTTCTCCCCC	ATACACATAAAACATATTTTTATT TTTTAGAAGGCAGAGA
antisense-*βFaar*	GATCACTAATACGACTCACTATAGGG ATACACATAAAACATATT	CGAGGAGGGGGTTCTCCCCCGCTC G
promoter-*βFaar*	GAGCTCTTACGCGTGCTAGCGCAGC ACAGCATTTAAAAAGGTAG	CAGTACCGGAATGCCAAGCTGTCC CCTCCCACCCCGGG
oe-Traf3ip2	AGAACATTTCTCTATCGATAATGAAC CGAAGCATTCCCG	ACCAACAGTACCGGAATGCCTCAC AAGGGTACCACCTGAAGG
oe-Smurf1	GACCTCCATAGAAGACACCGATGT CGAACCCCGGGACC	TAACGTTAGGGGGGGGGGAGTCA CTCCACTGCAAAGCCACA
βFaar bsp	TTTAATGAGAAAAATTATTTGGTTT	AACAAACTAAAACCCCTACCTC
Creb1-WT	TCGAGACCAGCAAGAAAAAGGAAG AAGGGTCAAAAAGTAGG T	CTAGACCTACTTTTTGACCCTTCTT CCTTTTTCTTGCTGGT C
Creb1-MUT	TCGAGCAACTACAGAAAAAGGAAG AAGGGTCAAAAAGTAGG T	CTAGACCTACTTTTTGACCCTTCTT CCTTTTTCTGTAGTTG C
Ins2-WT	TCGAGGTGGCATTGATCAGTGCTGC ACCAGCATCTGCTCCCTCTACCAGC TGGAGAACT	CTAGAGTTCTCCAGCTGGTAGAGG GAGCAGATGCTGGTGCAGCACTGA TCAATGCCACC
Ins2-MUT	TCGAGGTGGCATTGATCAGTGCTGA CAACTATTCTGCTCCCTCTACCAGC TGGAGAACT	CTAGAGTTCTCCAGCTGGTAGAGG GAGCAGAATAGTTGTCAGCACTGA TCAATGCCACC
NeuroD1-WT	TCGAGTAATTTAACTAATTTAAACC AGCAGAAAAGTGCTTAGAAAGTTA TTGCGTTGC T	CTAGAGCAACGCAATAACTTTCTA AGCACTTTTCTGCTGGTTTAAATTA GTTAAATTA C
NeuroD1-MUT	TCGAGTAATTTAACTAATTTAACAA CTACGAAAAGTGCTTAGAAAGTTAT TGCGTTGCT	CTAGAGCAACGCAATAACTTTCTA AGCACTTTTCGTAGTTGTTAAATTA GTTAAATTAC
βFaar-S1	GATCACTAATACGACTCACTATAGG GCGAGGAGGGGGTTCTCCC	GTGAGCAGGGATTTTCTATGTCAG TCTGGA
βFaar-S2	GATCACTAATACGACTCACTATAGG GAGTCCCTGCCTCAAGGAA	TTACTCTCCCAAAGGCCACAGACT TTTACA
βFaar-S3	GATCACTAATACGACTCACTATAGG GAATGACATTACTATCAGC	CATTGGTTCCTAACCTTCGTGAGCA TCAGG
βFaar-S4	GATCACTAATACGACTCACTATAGG GAGGGAAGGGTTCACCATT	AGAAGGCAGAGATCTACATCAAGG C
Phage-RIP vector	CGATGATAATATGGCCACACAT	ATCGATAGATCCTAATCAACCT

Table S5 (A) The antisense oligonucleotides (ASOs) and siRNA target sequences of *βFaar*.

siRNA name	5’-3’
si-1	GGTTTGAAGTGTTGTGTAT
si-2	GACTGTCAGTCCTGGAGAA
si-3	GTATGAGCAGAGAGCTATA
ASO-1	AAAAGCTGAGAAGCCAAGAC
ASO-2	GAACAGCCCACCTTTAAACA
ASO-3	GTATGAGCAGAGAGCTATA

(B) The sequence of siRNA or sgRNA

SiRNA/sgRNA name	5’-3’
si-Traf3ip2	GAACTCTAAGAACCAGCAA
si-Smurf1	GCTTCCTGCCCAGAGATAT
si-Dnmt3a	GCAGAACAAGCAGATGATT
si-Dnmt3b	CAGAGTATCAGGATGATAA
βFaar-sgRNA-1	AAAGTAGAAATGGTTAGCAG
βFaar-sgRNA-2	GCTCACTGTCAGTTTCTCTA
Has-βFAAR-sgRNA-1	GCGGCGGAGCACCGATCTCA
Has-βFAAR-sgRNA-2	CTACTCGTTTGGAACCGTTG

Table S6 The primers used in Real-time PCR (5'-3').

Gene	Forward Primer	Reverse Primer
βFaar	TGCCGCGGAGAGGATATTTTT	TGGGGTCTGGTAGACATCCT
Smurf1	AGCATCAAGATCCGTCTGACA	CCAGAGCCGTCCACAACAAT
Traf3ip2	TCCCGTGGAGGTTGATGAATC	TCAGGGTGCCTTCTAAAGAAACT
Gapdh	AGGTCGGTGTGAACGGATTTG	TGTAGACCATGTAGTTGAGGTCA
Nkx6.1	CTGCACAGTATGGCCGAGATG	CCGGGTTATGTGAGCCCAA
NeuroD1	ATGACCAAATCATACAGCGAGAG	TCTGCCTCGTGTTCCTCGT
Pdx-1	CCCCAGTTTACAAGCTCGCT	CTCGGTTCCATTCGGGAAAGG
Ins1	CACTTCCTACCCCTGCTGG	ACCACAAAGATGCTGTTTGACA
Ins2	GCTTCTTCTACACACCCATGTC	AGCACTGATCTACAATGCCAC
MafA	AGGAGGAGGTCATCCGACTG	CTTCTCGCTCTCCAGAATGTG
Creb1	AGCAGCTCATGCAACATCATC	AGTCCTTACAGGAAGACTGAACT
Glucagon	TTACTTTGTGGCTGGATTGCTT	AGTGGCGTTTGTCTTCATTCA
Has-βFAAR P1	TGCCCAATACCACATGGAC	AGATTTCCTTAAGCACATACTCC
Has-βFAAR P2	TCATGTCTGTTCCCAGCAA	TGAAGACTAATAGGCTCTGT
Has-βFAAR P3	GGGCATCTATTGTTCACCA	GAATCAGAAAGCCAACACC
Has-βFAAR P4	CTACTCGTTTGGAACCGTTG	AGAGCCCATAGGAATTACTCA
INSULIN	AGAGGCCATCAAGCAGATCACTGT	ACAGGTGTTGGTTCACAAAGGCTG

Table S7 The antibodies used in Western blot, IHC and IF.

Protein	Catalog	Dilution for WB	Dilution for IHC and IF
NKX6.1	CST (54551)	1:1000	
DNMT1	Abcam (EPR18453)	1:1000	
DNMT3A	Abcam (ab2850)	1:1000	
DNMT3B	Abcam (ab79822)	1:1000	
MAFA	CST (79737)	1:1000	
NEUROD1	CST (4373)	1:1000	
PDX-1	Proteintech (20989010AP)	1:1000	
BAX	Abcam (ab182733)	1:1000	
BCL-2	Abcam (ab182858)	1:1000	
HA-Tag	Abcam (ab9110)	1:1000	
TRAF3IP2	Abcam (ab137395)	1:1000	1:250
CREB1	CST (9197)	1:1000	
P65	Abcam (ab16502)	1:1000	
P-P65	Abcam (ab32536)	1:1000	
Pro-Caspase 3	Abcam (ab13847)	1:1000	
Cleaved Caspase3	Abcam (ab214430)	1:1000	
SMURF1	Abcam (ab236081)	1:1000	1:250
MnSOD	Abcam (ab13533)	1:1000	
Fas	Abcam (ab82419)	1:1000	
iNOS	Abcam (ab178945)	1:1000	
IκBα	Abcam (ab32518)	1:1000	
P-IκBα	Abcam (ab133462)	1:1000	
INSULIN	Abcam (ab181547)	1:1000	
INSULIN	Servicebio (GB13121)		1:500
GLUCAGON	CST (2760)		1:500
Argonaute 2	CST (2897s)	1:1000	
β-Actin	Abcam (ab8227)	1:1000	
goat anti-rabbit	santacruz blotechnology (L1015)	1:5000	
Goat polyclonal Secondary Antibody to Mouse IgG-H&L (Alexa Fluor® 647)	Abcam (ab150115)		1:1000
Goat	Abcam (ab150160)		1:1000

polyclonal Secondary Antibody to Rat IgG-H&L (Alexa Fluor® 594)			
